# Surveillance of acute lower respiratory infections in Cambodia: lessons from the field

**Published:** 2011-01-10

**Authors:** Sophie Goyet, Laurence Borand, Blandine Rammaert, Vantha Te, Patrich Lorn Try, Sopheak Hem, Bertrand Guillard, Philippe Buchy, Mardy Sek, Charles Mayaud, Sirenda Vong

**Affiliations:** 1 Epidemiology and Public Health Unit, Institut Pasteur in Cambodia, Phnom Penh, Cambodia; 2 Pediatric ward, Donkeo Hospital, Takeo, Cambodia; 3 Pediatric ward, Kompong Cham Hospital, Kompong Cham, Cambodia; 4 Biomedical Laboratory, Institut Pasteur in Cambodia, Phnom Penh, Cambodia; 5 Virology Unit, Institut Pasteur in Cambodia, Phnom Penh, Cambodia; 6 Pneumology and Intensive Care Unit, Hôpital Tenon, Paris, France

## Background

Acute lower respiratory infections (ALRI) are a leading cause of morbidity and mortality in developing countries. With the threat of emerging respiratory diseases, surveillance of viral respiratory infections has been an international focus of the recent years. In June 2007, we implemented in Cambodia a surveillance study of ALRI in two provincial hospitals. After 2 years of study, we highlight caveats and difficulties in interpreting surveillance data as a lesson from the field.

## Methods

Patients of all ages, hospitalized for acute lower respiratory infection (fever and cough and/or sore throat <14 days), were included in the surveillance study - excluding patients with known tuberculosis or positive HIV serology. For each patient, a medical exam and a chest –radiograph were systematically done. Blood, sputum, naso-pharyngeal swabs were collected on admission for culture molecular testing at Institut Pasteur- Cambodia. Diagnosis was performed by expert pulmonologists after reviewing patients’ medical and biological data and chest radiographs.

## Results

From April 2007 to December 2009, 3,566 subjects were enrolled. A diagnosis could be assigned by experts to 3,160 patients (88.6%). Among the 1,057 <5 year-olds, ALRI was confirmed for 961 (91%) patients (i.e. parenchymal, bronchial or pleural infection) and 96 (9%) presented with either upper respiratory infections (n=47) or extra-respiratory or non-infectious respiratory diseases (n=49). Among the 2,103 subjects aged 5 years and over, 1,882 (89.5%) had a confirmed ALRI, 10 (0.5%) presented with an upper respiratory infection, 211 (10%) an extra-respiratory or non-infectious respiratory disease. Among the <5 year-olds, a bacterial etiology could be determined in less than 1% of the ALRI cases - mainly due to the very low availability of sputum samples- while nasopharyngeal swabs were obtained in 99% of the children<5 year-olds. The virological positivity rates on nasopharyngeal swabs were higher in children <5y (57.3%) than in older children and adults (15.6%, p<0.001), but without any significant difference between patients with ALRI and patients with non-ALRI diseases, in both age-groups.

## Conclusion

Virological results provide an epidemiological picture of circulating and emerging viruses in the study population but causal associations between positive virology testing and ALRI have to be determined cautiously, taking into account bacteriological, clinical and radiographic data. Clinical data must include severity data and additional information such as time interval between symptoms onset and presentation, history of recent hospitalization or antibiotic intake, clinical evolution on treatment and time from the onset of symptoms.

